# Study of 1D and 2D Carbon Nanomaterial in Alginate Films

**DOI:** 10.3390/nano10020206

**Published:** 2020-01-24

**Authors:** Beatriz Salesa, Mar Llorens-Gámez, Ángel Serrano-Aroca

**Affiliations:** 1 Biomaterials and Bioengineering Lab, Centro de Investigación Traslacional San Alberto Magno, Universidad Católica de Valencia San Vicente Mártir, c/Guillem de Castro 94, 46001 Valencia, Spain; beatriz.salesa@ucv.es; 2 Escuela Técnica Superior de Arquitectura, Universitat Politècnica de València, Camí de Vera s/n, 46022 Valencia, Spain; malloga1@arq.upv.es

**Keywords:** graphene oxide, carbon nanofibers, calcium alginate, films, cytotoxicity, cell adhesion, human keratinocyte HaCaT cells

## Abstract

Alginate-based materials hold great promise in bioengineering applications such as skin wound healing and scaffolds for tissue engineering. Nevertheless, cell adhesion of mammalian cells on these hydrophilic materials is very poor. In cases such as polycaprolactone, poly(hydroxy-3-butyrate-co-3-valerate) and gelatin, the incorporation of hydrophobic carbon nanofibers (CNFs) and hydrophilic graphene oxide (GO) has shown significant improvement of cell adhesion and proliferation. The incorporation of these carbon nanomaterials (CNMs) into alginate films can enhance their mechanical performance, wettability, water diffusion and antibacterial properties. Herein, we report the effect of adding these CNMs into alginate films on cell adhesion for the first time. Thus, the results of this study showed that these nanocomposites are non-cytotoxic in human keratinocyte HaCaT cells. Nevertheless, contrary to what has been reported for other polymers, cell adhesion on these advanced alginate-based composites was not improved. Therefore, both types of composite films possess similar biological behavior, in terms of cell adhesion and non-cytotoxicity, and enhanced physical and antibacterial properties in comparison to neat alginate for potential biomedical and bioengineering applications.

## 1. Introduction

Alginate is a biodegradable, renewable, non-toxic, biocompatible and cost effective biopolymer composed of D-mannuronic and L-guluronic acid blocks [1,2]. This hydrophilic polymer has already been approved by the US Food and Drug Administration (FDA) for human biomedical applications [3]. Alginate hydrogels can be easily fabricated by crosslinking of sodium alginate (SA) with calcium cations [1]. These hydrophilic materials possess the special property of being able to retain large amounts of water, which renders them very promising for a wide range of bioengineering and biomedical applications such as wound healing, tissue engineering, controlled drug delivery and bioprocess engineering [4,5,6,7]. Nevertheless, it is well-known that cell adhesion of mammalian cells on alginate supports is very poor [1,4]. In this regard, very few material engineering approaches to enhance cell adhesion on alginate-based supports, such as alginate functionalization [8,9], have been successfully developed in the last decades. With respect to other biomaterials such as poly(hydroxy-3-butyrate-co-3-valerate) [10,11], the additions of low amounts (up to 1% *w/w*) of one-dimensional carbon nanofibers (CNFs) or two-dimensional graphene oxide (GO) nanosheets to produce advanced nanocomposites have also shown to be successful alternative strategies to enhance cell adhesion and proliferation. GO have also shown similar successful results in other important polymers such as polycaprolactone [12] and gelatin [13]. GO nanosheets possess outstanding physical properties like graphene [14] but easier processing, larger scale production and lower cost [15,16]. The 2D graphene oxide nanosheets possess oxygen-containing functional groups as hydroxyl (-OH) and carboxyl (-COOH) on the basal planes and at the edges, which render them hydrophilic and soluble in water [14]. However, CNFs are highly hydrophobic and non-polar in nature [17]. These filamentous carbon nanomaterials (CNMs) possess excellent physical properties and, in comparison with GO, they have lower price and higher electrical conductivity [18]. Thus, CNFs can be utilized to fabricate conductive composites [19] for biomedical applications [20].

Alginate films, like most hydrophilic polymers, exhibit poor mechanical performance when they are hydrated at body temperature (~37 °C) in biomedical applications. Thus, the mechanical properties of these polymer networks can be improved following a broad range of established reinforcing methods developed for polymers: reinforcement by interpenetrating polymer networks (IPNs) [21], rise of crosslinking density [22,23], addition of nanofibers [24,25], plasma polymerization methods [26,27] and more recently, by the incorporation of low contents (up to 1% *w/w*) of CNMs such as 1D CNFs or 2D GO nanosheets [28,29,30,31,32,33]. In addition, this nanotechnological approach was able to enhance other physical and biological properties of calcium alginate films such as wettability, water diffusion [29,30] and antibacterial activity against the life-threatening methicillin-resistant *Staphylococcus epidermidis* [34,35]. Nevertheless, no studies of cell adhesion on calcium alginate/CNFs or calcium alginate/GO films have been reported so far in literature. Therefore, according to the previous successful results of cell adhesion reported for other hydrophobic and hydrophilic polymers [11,12,13], we hypothesized here that the incorporation of the same low-cost amounts (up to 1% *w/w*) of these CNMs into calcium alginate films will be able to enhance cell adhesion without causing any cytotoxic effect in human keratinocyte HaCaT cells. The use of a low amount of CNMS, which are currently expensive materials, is important to reduce production costs as much as possible and modify the chemical nature of the alginate biopolymer as less as possible. Since alginate-based biomaterials have many applications in skin wound care and in the fabrication of skin scaffolds [36,37,38,39], keratinocyte cells were used to demonstrate the ability of the calcium alginate/CNFs and calcium alginate/GO membranes to support epidermal cell adhesion.

## 2. Materials and Methods

### 2.1. Materials

Sodium alginate (Panreac AppliChem GmbH, Darmstadt, Germany), calcium chloride as crosslinker (≥93.0%, Sigma-Aldrich, St. Louis, MO, USA), graphene oxide nanosheets (Product Code: 796034, 15–20 sheets, 4% to 10% edge-oxidized, Sigma-Aldrich, USA) and carbon nanofibers (Product Code: 13/0248, Graphenano, Yecla, Spain) were utilized as supplied.

### 2.2. Alginate-Based Film Preparations

Calcium alginate films were prepared with 0%, 0.1%, 0.5% and 1% *w/w* of CNFs or GO nanosheets (with respect to the mass of SA). Thus, the required CNFs or GO contents were dispersed in 22 mL of distilled water. Subsequently, 0.25 g of SA were dissolved in this CNFs/water or GO/water dispersion by magnetic stirring for 1 h at ambient temperature (24 ± 0.5 °C). After that, 0.0159 g of calcium chloride dissolved in 10 mL of distilled water was mixed with the CNFs/SA or GO/SA aqueous solutions under magnetic stirring for 10 min to be directly poured into Petri dishes and left at 37 °C for 24 h to fabricate thin films by solvent evaporation. The produced films were immersed into 2% *w/v* aqueous CaCl_2_ solution for 2 h and rinsed three times after that with distilled water to be finally vacuum dried at 60 °C ± 0.5 °C (to constant weight). These samples will be hereafter referred to as 0.1%CNFs, 0.5%CNFs, 1%CNFs, 0.1%GO, 0.5%GO and 1%GO according to the weight percent of CNFs or GO incorporated into the calcium alginate films. The control sample of calcium alginate produced with 0% of CNMs will be hereafter named as CA0%.

### 2.3. Characterization

#### 2.3.1. Alginate Characterization

The SA utilized in this study was characterized by Size Exclusion Chromatography with Multi Angel Light Scattering (SEC-MALS) detection, high performance anion-exchange chromatography with pulsed amperiometric detection (HPAEC-PAD) and nuclear magnetic resonance (NMR) spectroscopy by the NOBIPOL group at the NTNU Norwegian University of Science and Technology.

#### 2.3.2. Raman Spectroscopy and Electron Microscopy

Raman continuous scans from 1000 to 3000 cm^−1^ was carried out in a Renishaw inVia confocal micro-Raman apparatus at 600 L·mm^−1^ grating utilizing an argon ion laser at 633 nm edge (power 10%), ×20 lens, Renishaw CCD camera detector. The CNFs and GO nanosheets in pure form and incorporated into the calcium alginate nanocoposite films were placed onto glass disks for direct analysis. The morphologies of the GO nanosheets and CNFs were observed by high-resolution transmission electron microscopy (HR-TEM) in a JEM 2100F 200 kV apparatus (JEOL, Japan). This electron microscope is equipped with energy-disperse X-ray spectroscopy (EDS) for C/O ratio estimation at 20 kV. The GO and CNFs powders were dispersed in dichloromethane for 10 min and subsequently dried at ambient temperature (24 ± 0.5 °C) before microscopic observation. An estimation of the length and diameter of the CNFs as mean values ± standard deviation was calculated by measuring the length and diameter of 50 carbon nanofibers. The average lateral dimension of the utilized GO was estimated by measuring the lateral length of 50 nanosheets and expressed as mean values ± standard deviation. The cross section, produced by cryogenic fracture, and surfaces of the calcium alginate/GO and calcium alginate/CNFs composites were observed by field emission scanning electron microscopy (FESEM) at a magnification of 1000x and 11160x, respectively, utilizing a ZEISS Ultra 55 Model microscope (Carl Zeiss SMT GmbH, Oberkochen, Germany) at 2 kV.

#### 2.3.3. Cytotoxicity and Cell Adhesion Assays

MTT cytotoxicity tests according to the ISO-10993 were performed with the extracts of films (10-mm diameter) using a volume ratio of 3 cm^2^/mL. The specimens were sterilized under ultraviolet light per each side for 1 h. The films (*n* = 4) were located into a 12-well plate containing 1 mL of DMEM (Biowest SAS, Nuaillé, France) without Fetal bovine serum (FBS) per well to ensure complete covering of the whole surface area of each sample film. Cell incubations were always performed in a humidified air atmosphere with 5% CO_2_ for 72 h at 37 °C. Thus, the extracts were collected after incubation and filtered through 0.20 µm pores to be used immediately for the cytotoxic tests. The MTT tests were performed with human keratinocyte HaCaT cells provided by the Medical Research Institute Hospital La Fe, Spain. These type of cells, which were non-tumorigenic and immortalized, were incubated (5% CO_2_) in DMEM with 10% FBS (Biowest SAS, Nuaillé, France) mixed with 100 units/mL penicillin (Lonza, Belgium) and 100 mg/mL streptomycin (HyClone, GE Healthcare Life Sciences, Issaquah, WA, USA). The toxic effect of the film extracts on cell viability were determined on 96-well plate with cells planted at 5·10^5^ cells per well. After incubation for 24 h, 100 µL of medium of each well was replaced with 100 µL of film extracts. As control results, the medium was replaced with 100 µL of the medium used to produce the film extracts (negative control,) and 100 µL of 1000 µM zinc chloride (≥97.0%, Sigma Aldrich, St. Louis, MO, USA) cytotoxic solution (positive control) [34]. Cell incubation was performed with 5 mg/mL MTT per well for 4 h. A volume of 100 µL of dimethyl sulfoxide (DMSO, Sigma Aldrich, St. Louis, MO, USA) was used to dissolve the formazan crystals at ambient temperature. Finally, absorbance measurements were taken at 490 nm on a microplate reader (Varioskan, Thermo Fisher, Waltham, MA, USA).

Cell adhesion was studied by fluorescence microscopy [40] in a Motic BA410 ELITE Series microscope equipped with a Complete EPI-Fluorescence Kit Motic. Human keratinocyte HaCaT cells were provided by the Medical Research Institute Hospital La Fe, Valencia, Spain. The films were cleaned with absolute ethanol and sterilized using ultraviolet light per each side for 1 h. Thus, the films were hydrated in a 24 multiwell plate (1 disk per well) during 30 min using 0.5 mL DMEM without FBS. After hydration, cells were seeded at a density of 2·10^4^ cells per well onto the different film surfaces. Round glass coverslips were used as control samples. After cell incubation for 24 h, each well was rinsed with PBS. Thus, the cells were fixed with PFA 4%, permeabilized with 0,1% Triton X-100 in PBS, blocked with 10% FBS in PBS and stained with Phalloidin-FITC for 40 min and DAPI for 5 min. Finally, the films were protected using the mounting medium Fluoromount (Sigma Aldrich, St. Louis, MO, USA) to preserve fluorescence. The Phalloidin-FITC, DAPI and Fluoromount compounds were all purchased from Sigma-Aldrich. For cell quantification analysis, DAPI nuclei staining of 5 random fields covering the entire films per group were quantified and normalized to the total counted area in triplicates in three independent experiments. Numbers of DAPI stained cell nuclei counted on the material surfaces were expressed as percentage of change on any of the conditions versus glass coverslips (considered 100%).

The cytotoxicity and cell adhesion results were statistically analyzed by ANOVA followed by multiple Tukey’s post-hoc by the GraphPad Prism 6 software at significance level of at least *p* < 0.05.

## 3. Results

### 3.1. Alginate Characterization

The weight-average (M_w_) and number-average (M_n_) molecular weights of the SA used in this study were 379.5 ± 9.5 and 170.7 ± 3.1 KDa, respectively. More characterization information and the ^1^H-NMR spectrum (Figure S1) of the commercial sodium alginate used in this study are available in the Supplementary Materials.

### 3.2. Raman Spectroscopy and Electron Microscopy

**Figure 1:**
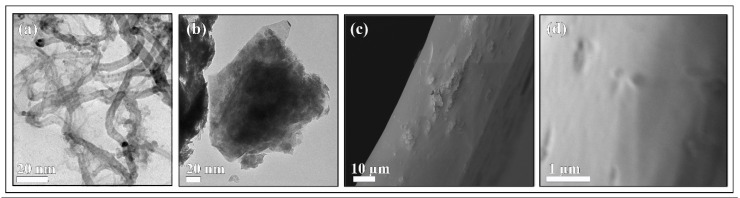
Electron microscopy images: (**a**) HR-TEM of one-dimensional carbon nanofibers, (**b**) HR-TEM of two-dimensional graphene oxide nanosheets, FESEM of (**c**) cross section (at 1000x) and (**d**) surface (at 11160x) of calcium alginate composite films with 1% *w/w* of graphene oxide.

The GO nanosheets employed in this study can be classified according to the number of graphene layers, average lateral size and carbon-to-oxygen (C/O) atomic ratio determined by Raman spectroscopy and HR-TEM equipped with EDS according to the GRAPHENE Flagship Project of the European Union for the unequivocal classification of these materials [41]. The Raman spectra of the CNFs and GO nanosheets are shown in Figure S2 in the Supplementary Materials. Raman spectroscopy provides valuable structural information of carbon nanomaterials such as CNFs [42] and GO [14]. Thus, the Raman scans of the GO nanosheets showed the typical D and G bands at approximately 1330 and 1580 cm^−1^, respectively, and the 2D band at ~2660 cm^−1^ as expected [43,44]. The *D band intensity/G band intensity* ratio (I_D_/I_G_ ratio) was 0.92 for this type of GO, which is related with the defect/disordered ratio of carbon nanostructures [45,46]. The intensity of the Raman peaks of GO suggests that the GO layers are not single layers. Since the intensity of the G peak is known to potentially increase with increasing the number of layers [47], the I_2D_/I_G_ ratio of 0.87 determined in Figure S2 corresponds to a number of GO layers > 10 in good agreement with the product information provided by the manufacturer (Sigma-Aldrich). On the other hand, the Raman spectrum of the CNFs showed also the D, G and 2D bands at similar Raman shifts to those observed for GO. However, the I_D_/I_G_ ratio was 1.51 due to these carbon filamentous materials possessing a higher degree of disorder, which is typical of irregular carbon structures [42]. The Raman analysis of the calcium alginate/GO and calcium alginate/CNFs nanocomposite films exhibited the same bands as those shown for GO and CNFs, respectively. However, the intensity of these bands was much lower due to the small amount of carbon nanomaterials present in the nanocomposite films (images not shown). The HR-TEM images showed that the CNFs are one-dimensional hollow filaments with a wide range of diameters (22.7 ± 11.9 nm) and lengths (737.8 ± 522.4 nm) (see Figure 1a).

However, the GO exhibited a morphology of 2D nanosheets with average lateral dimension of 153.8 ± 57.2 nm adhered by van der Waals forces with residual π–π stacking between faces as expected [14] (see Figure 1b). The EDS results of CNFs and GO showed C/O ratios of 31.3 and 15.4, respectively. The morphology of the calcium alginate/GO nanocomposite film prepared with the highest GO content (sample 1%GO) is shown in Figure 1c,d. These FESEM micrographs of cross section and surface show clearly how the GO nanosheets are embedded into the alginate polymer matrix. The morphology of the other composite films with lower GO content or any CNFs content does not differ much from that of 1%GO (images not shown).

### 3.3. Cytotoxicity and Cell Adhesion

**Figure 2:**
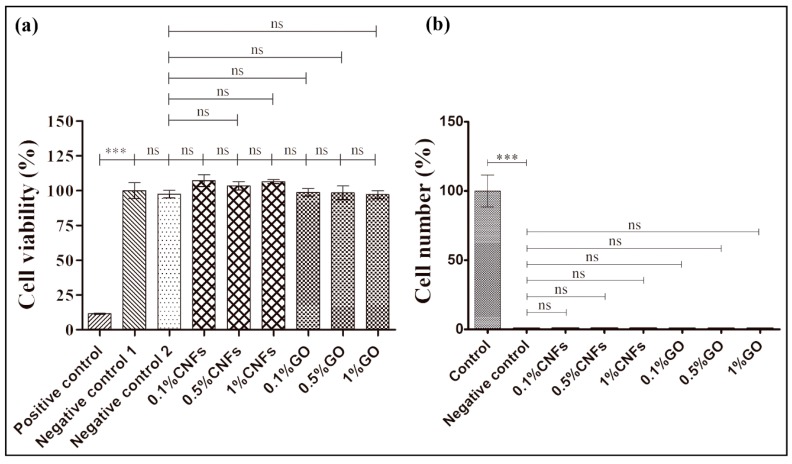
MTT cytotoxicity results (% of cell viability of human keratinocyte HaCaT cells) for the extracts of the calcium alginate films with 0.1%, 0.5% and 1% *w/w* of CNFs or GO nanosheets, negative control 1 (medium), negative control 2 (calcium alginate without carbon nanomaterials) and positive control (zinc) (**a**), and cell adhesion quantification results by DAPI staining (**b**) on glass coverslips (control) and calcium alginate with 0 (negative control), 0.1%, 0.5% and 1% *w/w* of CNFs or GO nanosheets. Data was expressed as mean ± SD and compared by ANOVA-Tukey’s post-hoc: *** *p* > 0.01; ns: not significant.

The addition of these CNMs into alginate films can produce a significant enhancement of mechanical performance, wettability, water diffusion and antibacterial activity [28,29,30,34,35]. Herein, we report the effect of incorporating these CNMs into alginate films on cytotoxicity and cell adhesion. Thus, the MTT cytotoxicity assay performed with human keratinocyte HaCaT cells showed no statistically significant differences (~100% viability) between the negative controls (medium and extract of the CA0% sample) and the extracts of the composite films (Figure 2a).

**Figure 3:**
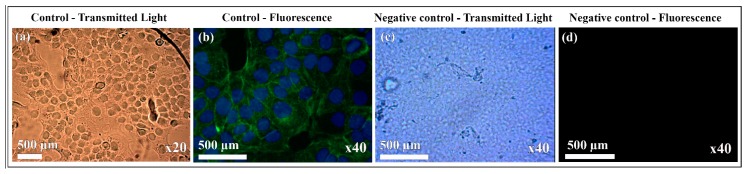
Cell adhesion of human keratinocyte HaCaT cells on glass coverslips (control) (**a**,**b**) and on calcium alginate films without carbon nanomaterials (negative control) (**c**,**d**) by optical microscopy in transmission light mode and fluorescence microscopy (DAPI and Phalloidin-FITC staining). The magnification of the utilized objective lens is indicated for each image. Cell adhesion on the glass coverslips is observed by either optical or fluorescence microscopy. Nevertheless, no cell adhesion is observed by neither optical nor fluorescence microscopy on calcium alginate.

Therefore, the alginate films with CNFs or GO contents ranging from 0% to 1% *w/w* are non-cytotoxic in human keratinocyte HaCaT cells. On the other hand, the results of this study showed good adhesion of human keratinocyte HaCaT cells on the glass coverslips (control) and no cell adhesion on the calcium alginate films (negative control) as expected (see Figure 3). Thus, the quantification of the nuclei stained with DAPI showed 0% of cell adhesion on calcium alginate (see Figure 2b) in comparison with the glass coverslips (considered 100%).

**Figure 4:**
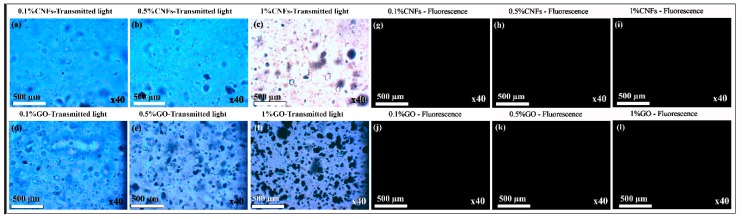
Cell adhesion results of human keratinocyte HaCaT cells on calcium alginate films with 0.1%, 0.5% and 1% *w/w* of CNFs and GO by optical microscopy in transmission light mode (**a**–**f**) and fluorescence microscopy (DAPI and Phalloidin-FITC staining) (**g**–**l**). The magnification of the utilized objective lens is indicated for each image. No cell adhesion on any of the composite films was observed by either optical or fluorescence microscopy.

However, contrary to our hypothesis, the results of this study showed no cell adhesion of human keratinocyte HaCaT cells on the alginate-based composite films independently of the CNFs or GO concentration (see Figure 4). Thus, the quantification of the nuclei stained with DAPI showed also 0% of cell adhesion (in Figure 2b) on the composite films and thus no increased number of keratinocyte cells in comparison with the calcium alginate film (negative control).

Therefore, in spite of the previous successful results achieved with other polymers of different chemical nature [10,11,12,13], the incorporation of low CNFs or GO contents into calcium alginate films was not able to improve the cell adhesion of human keratinocyte HaCaT cells. Carbon nanomaterials can be crosslinked through the interaction of their oxygen-containing functional groups with divalent cations such as Ca^2+^ by coordination chemistry [43,48,49,50]. For that reason, the divalent cations of calcium can simultaneously crosslink alginate chains and the CNMs producing strong 3D composite networks [29,30]. Therefore, in spite of their different chemical nature, both one-dimensional hydrophobic CNFs and two-dimensional hydrophilic GO nanosheets remain embedded in the alginate polymer matrix and, therefore, not exposed to enhance cell adhesion on the composite surface, in good agreement with the FESEM observations (see Figure 1). Nonetheless, these composite films show similar promising applications to those of calcium alginate films with enhanced physical and antibacterial properties [28,29,30,34,35]. Therefore, these alginate/CNFs and alginate/GO composite films are very promising for a broad range of bioengineering and biomedical applications.

## 4. Conclusions

This study provides evidence that the combination of nanotechnology with polymer engineering is not always a successful strategy to enhance cell adhesion on polymer films, contrary to what has been previously reported. Thus, the incorporation of 1D hydrophobic carbon nanofibers and 2D hydrophilic graphene oxide nanosheets into alginate films showed no cytotoxicity but did not enhance cell adhesion of human keratinocyte HaCaT cells. This result can be attributed to the fact that Ca^2+^ ions are able to simultaneously crosslink the carbon nanomaterials and alginate biopolymer chains producing tight composite surfaces with the one-dimensional and two-dimensional nanomaterials not exposed to cells. Nevertheless, the demonstrated non-cytotoxicity of these composite films, which possess superior physical and antibacterial properties, broadens the future applicability of alginate films in biomedicine and bioengineering. Nonetheless, challenges remain, and further research in this field could reveal different chemical engineering routes able to produce different morphologies and/or compositions with enhanced cell adhesion properties, which are highly desirable for certain biomedical applications such as tissue engineering.

## Supplementary Materials

The following are available online at https://www.mdpi.com/2079-4991/10/2/206/s1. Figure S1: 1H-NMR spectrum of the partly hydrolyzed sodium alginate: anomeric area (a) and full 1H-NMR spectrum (b), Figure S2: Raman continuous scans of one-dimensional carbon nanofibers (a) and two-dimensional graphene oxide nanosheets (b).
