# Supplementary material for: Study of 1D and 2D Carbon Nanomaterial in Alginate Films
Source: Nanomaterials (Basel). 2020 Jan 24;10(2):206. doi: 10.3390/nano10020206 (PMC7074849; doi:10.3390/nano10020206)
Supplement: Supplementary file 1 [file nanomaterials-10-00206-s001.pdf]

nanomaterials

Supplementary materials

Study of 1D and 2D Carbon Nanomaterial in Alginate Films

Beatriz Salesa[1], Mar Llorens-Gámez[2], Ángel Serrano-Aroca[1,*]

[1]Biomaterials and Bioengineering Lab, Centro de Investigación Traslacional San Alberto Magno, Universidad Católica de Valencia San Vicente Mártir, c/Guillem de Castro 94, 46001 Valencia, Spain; beatriz.salesa@ucv.es

[2]Institute for Research and Innovation in Bioengineering, Universitat Politècnica de València, Camí de Vera s/n, 46022 Valencia, Spain; malloga1@arq.upv.es

* Correspondence: angel.serrano@ucv.es; Tel.:+34-963637412 (Ext:5256)

Supplementary Materials

1. Alginate characterization.

The percentages of guluronic acid, guluronic acid in blocks of dimers and guluronic acid in blocks of trimmers were 43%, 27% and 23% respectively. The measured ^{1}H-NMR spectrum of the sodium alginate utilized in this study is shown in Figure S1.

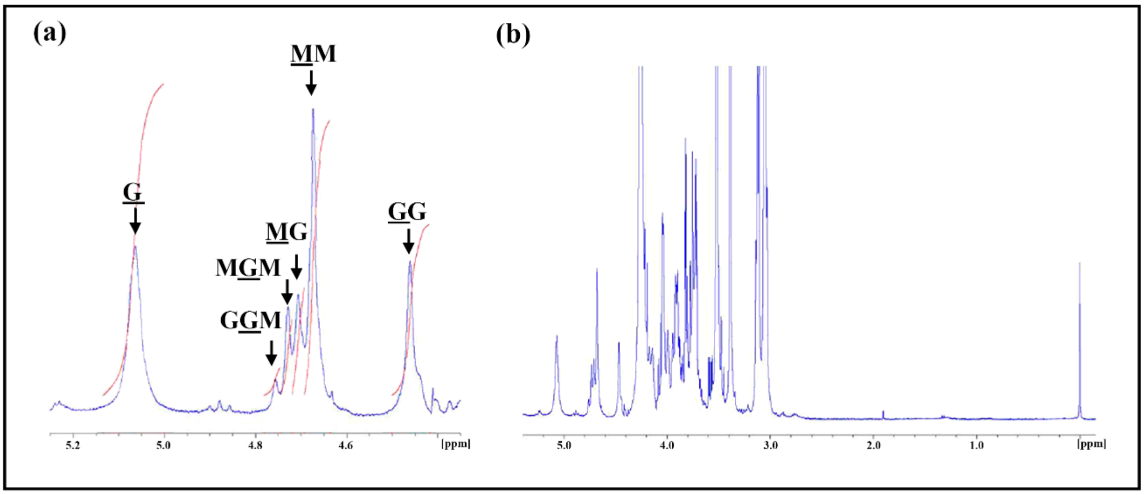

Figure S1. ^{1}H-NMR spectrum of the partly hydrolyzed sodium alginate: anomeric area (a) and full ^{1}H-NMR spectrum (b).

2. Characterization of the carbon nanomaterials.

The Raman spectra of the two carbon nanomaterials used in this study, one-dimensional carbon nanofibers and two-dimensional graphene oxide nanosheets, are shown in Figure S2.

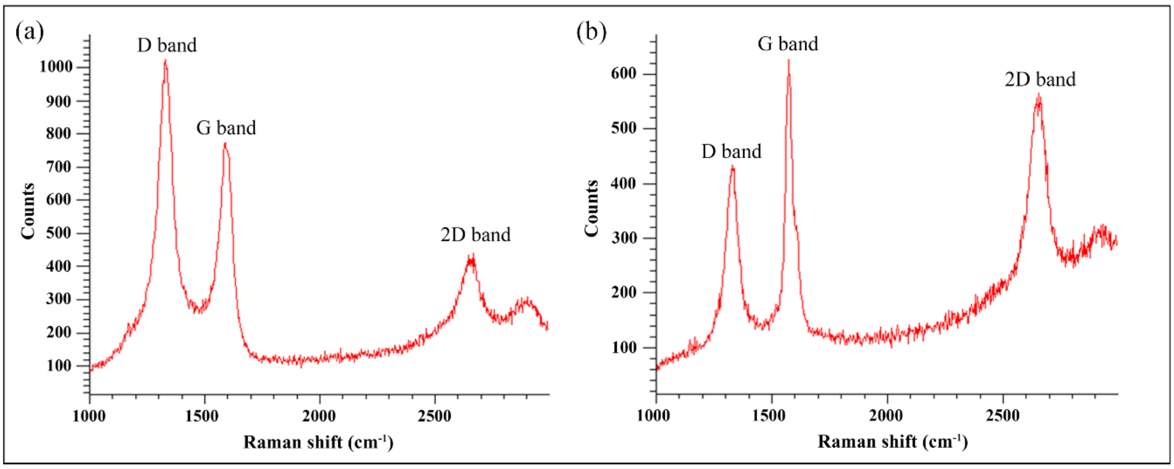

Figure S2. Raman continuous scans of one-dimensional carbon nanofibers (a) and two-dimensional graphene oxide nanosheets (b)

Author Contributions: Conceptualization: Á.S-A; Data curation: Á.S-A; Formal analysis: Á.S-A; Funding acquisition: Á.S-A; Investigation: B. S, M. Ll-G, Á.S-A; Methodology: Á.S-A; Project administration: Á.S-A; Resources: Á.S-A; Software: Á.S-A; Supervision: Á.S-A; Validation: Á.S-A; Visualization: Á.S-A; Roles/Writing - original draft: Á.S-A; Writing - review & editing: Á.S-A.

Funding: This research was funded by FUNDACIÓN UNIVERSIDAD CATÓLICA DE VALENCIA SAN VICENTE MÁRTIR through the grant number 2019-231-003UCV.

Acknowledgments: The authors would like to acknowledge Professor Finn Lillelund Aachman and his research team from the NOBIPOL group at the NTNU Norwegian University of Science and Technology for the characterization of the sodium alginate employed in this work.

Conflicts of Interest: The authors declare no conflict of interest.

© 2020 by the authors. Submitted for possible open access publication under the terms and conditions of the Creative Commons Attribution (CC BY) license (http://creativecommons.org/licenses/by/4.0/).
